# High-Throughput Method of Whole-Brain Sectioning, Using the Tape-Transfer Technique

**DOI:** 10.1371/journal.pone.0102363

**Published:** 2015-07-16

**Authors:** Vadim Pinskiy, Jamie Jones, Alexander S. Tolpygo, Neil Franciotti, Kevin Weber, Partha P. Mitra

**Affiliations:** 1 Cold Spring Harbor Laboratory, Cold Spring Harbor, New York, United States of America; 2 Department of Biomedical Engineering at Stony Brook University, Stony Brook, New York, United States of America; University of Florida, UNITED STATES

## Abstract

Cryostat sectioning is a popular but labor-intensive method for preparing histological brain sections. We have developed a modification of the commercially available CryoJane tape collection method that significantly improves the ease of collection and the final quality of the tissue sections. The key modification involves an array of UVLEDs to achieve uniform polymerization of the glass slide and robust adhesion between the section and slide. This report presents system components and detailed procedural steps, and provides examples of end results; that is, 20μm mouse brain sections that have been successfully processed for routine Nissl, myelin staining, DAB histochemistry, and fluorescence. The method is also suitable for larger brains, such as rat and monkey.

## Introduction

A basic component of the neuroanatomical workflow is efficient and reliable histological processing; and subsequent analysis and correct interpretation of experimental results heavily depend on high-quality histological sections. Ease of processing is another factor, especially in the very common case where processing is being carried out by students or non-expert technical staff. In this report, we describe a protocol for tape-assisted cryosectioning which significantly improves section quality and the speed of collecting brain sections.

**Fig 1:**
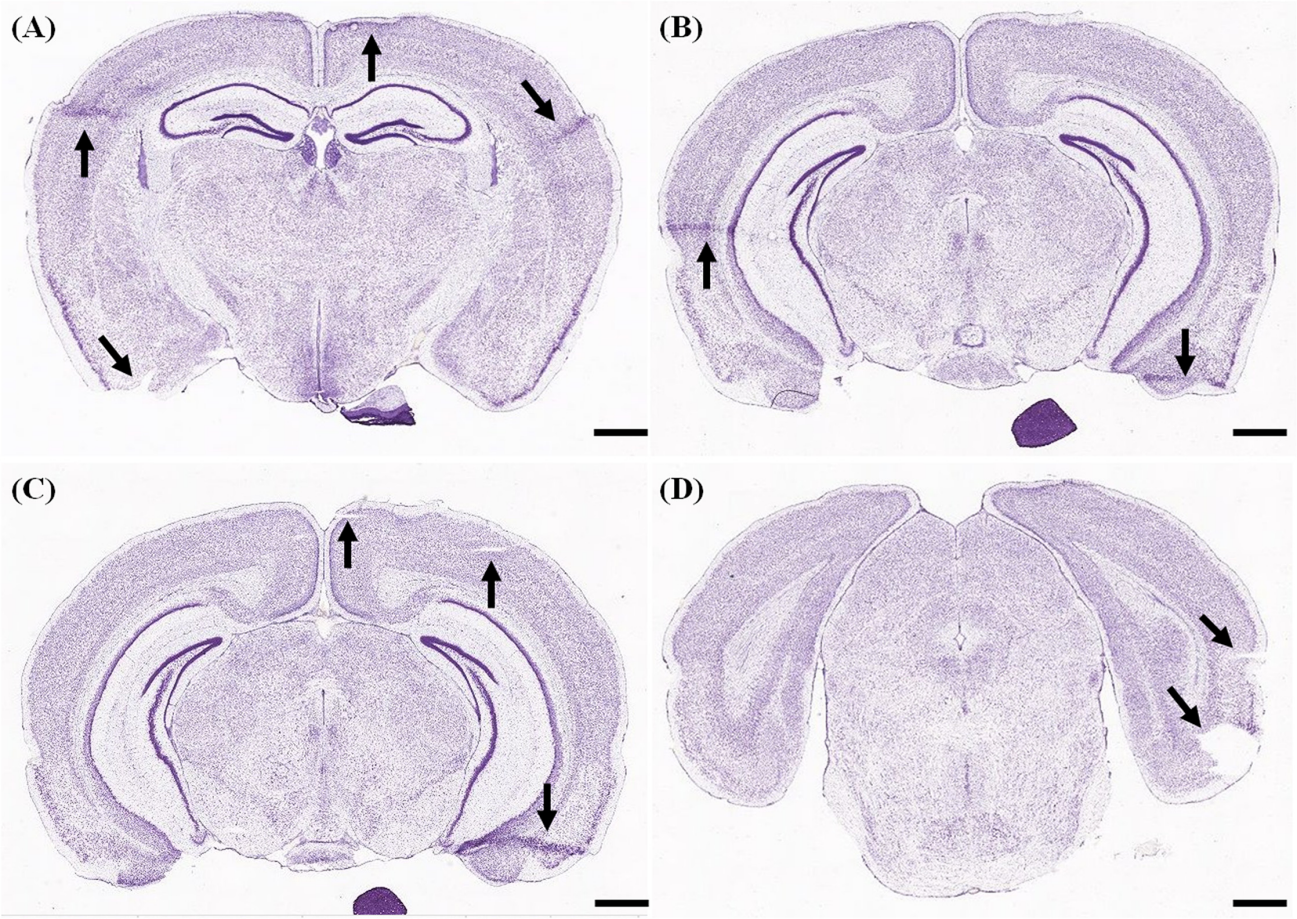
Common examples of section damage (downloaded from the Allen Reference Atlas). Obtaining undamaged cryostat sectioned material is difficult, especially for relatively thin sections (20–25μm). A scan through the Allen Reference Atlas, one of the most commonly used anatomical references of the mouse, turns up examples of section damage (arrows). The three most common sources of damage are: folded areas (solid black arrows), as in A, B and C; torn sections (arrowheads, as in C), and sections with missing areas (dotted arrows, in D). Allen Reference Atlas, coronal Level 73 is shown in (A); level 83 is shown in (B); level 84 is shown in (C); and level 96 is shown in (D). Scale bars are 1mm.

Cryostat tissue sectioning is a popular means of sectioning brain tissue for further processing, imaging, and analysis. It is, however, labor-intensive and even for expert users, there is some risk of lost or damaged sections (Fig 1). In conventional cryostat sectioning, there are many methods of collecting individual sections as these are cut (“shaved”) off the main block of tissue. Two basic such methods are: (1) using a fine brush to transfer sections to solution for further, free-floating processing or for mounting on slides, (2) directly pressing sections onto glass slides from the anti-roll plate of the cryostat. The second method has the advantage of minimizing the degree of section handling, but will result in torn, wrinkled, or curled sections unless the temperature differential between the sections and pick-up slide is optimal.

A commercially available tape system (CryoJane; Instrumedics/Leica Microsystems) has been developed to facilitate the transfer of cut sections from the blockface to the slide. In this procedure, adhesive tape is attached to the blockface, the block is sectioned without the standard anti-roll plate, and the detached, cut section remains adhered to the tape without curling or other deformation. The tape and section together are placed onto a polymer-coated glass slide that polymerizes under ultraviolet (UV) light. After polymerization, the section binds with the slide and the overlying tape can easily be peeled off.

We have made several hardware and procedural modifications to the CryoJane product that resulted in a significantly enhanced sectioning system. These are: (1) the use of an array of UV-LEDs to increase the amount of UV energy that is applied to the polymer coated glass slides, (2) the fabrication of a curing platform with two such UV-LED arrays, which allows for the convenient collection of alternating sections for parallel processing in two slide series, (3) determining optimal parameters for tissue preparation and sectioning temperatures. These modifications have allowed for 2-3X greater productivity and a significant decrease in the number of damaged sections. To our knowledge, this is the first use of the tape-transfer method for whole-brain sectioning of perfused mouse tissue, where all cut sections are mounted, stained, imaged and evaluated. We show that this technique can be used to consistently produce high-quality histological sections that are anatomically preserved in relation to the blockface. We also show that tape-transfer cut sections can be used both for the detection of native fluorescence and for histochemical processing.

## Materials and Methods

Three basic steps were required for cutting and mounting frozen mouse brain sections without introducing distortions or section damage. In brief, these are: (1) Section collection onto tape. For each section to be collected, an individual segment of tape (“tape-window”) is manually attached to the face of the tissue block, using a hard-roller. The tape remains attached to the section, as it is cut from the block, and helps to maintain the shape and integrity of the section. (2) Section transfer to slide. The tape and adhered section are transferred to a curing platform and placed onto a slide that is pre-coated with a UV-activated polymer. Air bubbles are removed using a soft-roller. This process is repeated for up to 3 tape windows per slide. (3) UV curing of the sections. The lever door of the platform is then closed, and a brief pulse of UV light is applied. This results in curing of the UV polymer and a firm adhesion of the sections to the slide. All tape-windows are then peeled away and the slides are removed from the cryostat.

### 2.1 System Components

In addition to the mouse brains, system components are: 1 curing platform with 2 UV-LED arrays; 1 hard-roller; 1 soft-roller; multiple tape-windows, glass slides, and a cryostat. The number of tape-windows and glass slides depends on the size of the sample to be cut. One tape-window is needed for each section that is collected. Up to 3 tape-windows can be placed on a single 1X3 slide. A comparison of this system and the commercially available CyoJane is shown in Table 1. All components were customized for this application, as detailed below.

**Table 1:** Customized Parameters of Tape-Transfer Curing Platform.

	Commercial CryoJane	Modified System
**Duration of UV pulse**	8 milliseconds	~5–6 seconds (can be increased as needed)
**Number of pulses required**	>2 (slide is rotated between pulses)	1
**Interval between repeated pulses**	>30 seconds (or fuse will blow)	0.00
**Total Irradiance per pulse (uW-sec)**	40	**1600–2000**
**Slide Capacity**	1	**2**
**Fuse Replacement**	2-5x per week (continuous use)	No Fuses
**Sectioning Rate (Slides/Hour)**	20	**50**

The use of the UV-LEDs as a means of adhering sections to the slide is superior to the commercial CryoJane system because the UV can be applied over a longer time (seconds versus milliseconds). This extended application time allows in a 40–50X increase in the total amount of UV that is applied to the slide. We have arranged the UV-LEDs into an array that delivers equal amount of UV across the entire slide. Therefore a single pulse of UV can be used to adhere all of the sections on the slide, without the need to rotate the slide. As a result of these improvements, sectioning rate has increased by 2.5X. Section quality is system independent (when 2 pulses per slide are used with the CryoJane system) and a direct function of sectioning procedure (Fig 3).

#### 2.1.1 Animals

All animal studies, experiments, and procedures were discussed and approved by the Institutional Animal Care and Use Committee at Cold Spring Harbor Laboratory, and conform to all federal regulations and the NIH Guidelines for the Care and Use of Laboratory Animals. All animals were acquired from Jackson Laboratories (stock 000664). The animals were 56±3 day old mice, C5BL/6, with a weight of 18.8–26.4 grams. Avertine (2.5%) was used as the anesthetic. The animals were perfused with 4% paraformaldehyde (PFA; JT Baker, JTS898-7), after a saline preflush of 50mL that was used to remove the blood. The brains were extracted and post-fixed in a solution of 4% PFA with 10% sucrose (JT Baker, 4072–05) in PBS, for 24 hours. The brains were further cryo-protected in 20% sucrose in PBS for an additional 24 hours.

#### 2.1.2 Curing Platform

**Fig 2:**
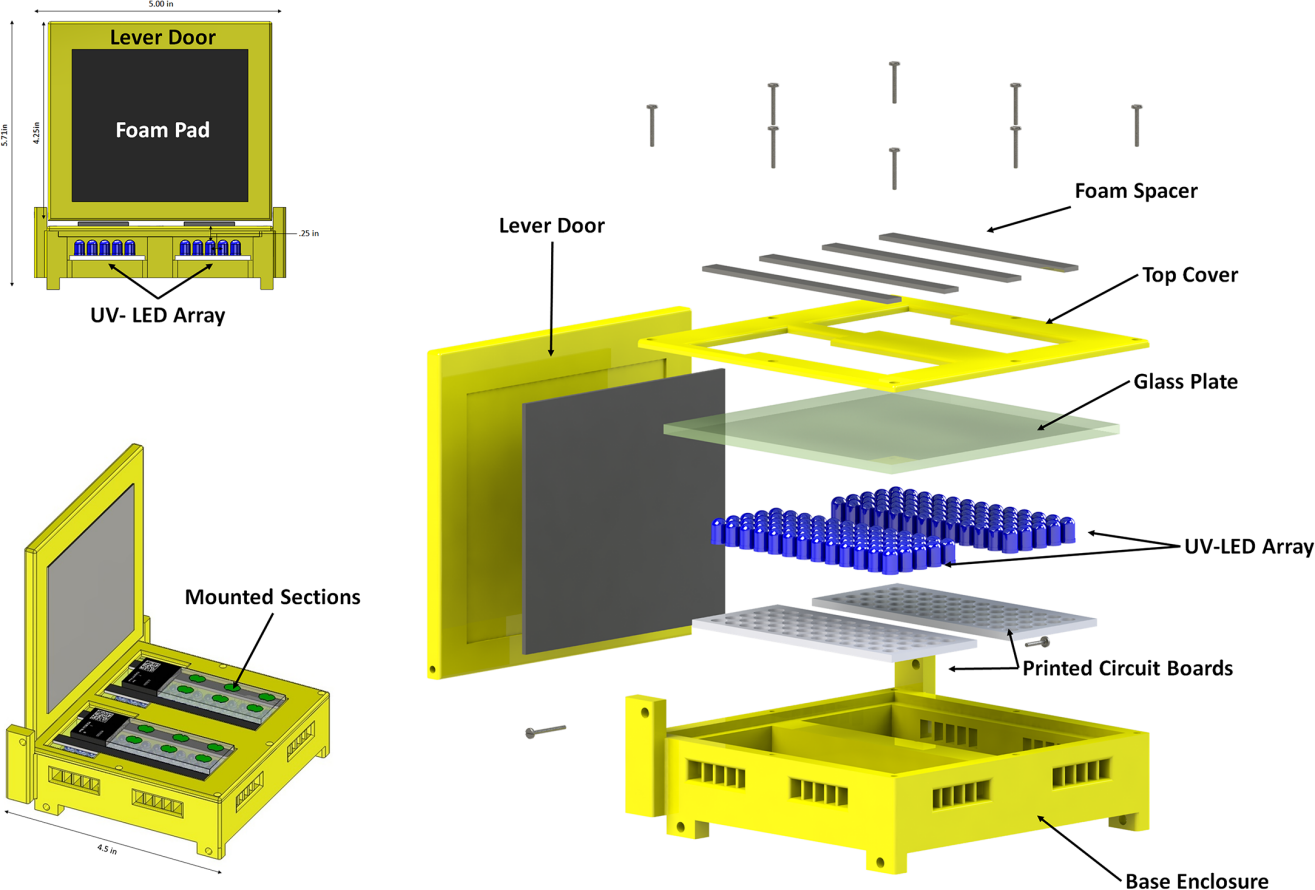
An exploded-view of the redesigned tape-transfer system curing platform. The platform is composed of: the base enclosure, the printed circuit board (PCB) with soldered UV-LEDs, a glass plate, a top cover, foam spacers, a foam pad, and a lever door. With the exception of the PCB, the glass plate, the foam spacers and pad, all other components were 3-D printed in VeroWhite, through Vistatek Corporation (Vadnais Heights, MN). The base enclosure has side access, to allow for connecting the ground and power terminals of the UV-LEDs. The enclosure as illustrated at bottom left allows for sections to be collected on two slides and for the slides to be cured simultaneously. A front view of the platform is shown on the top left. When lowered, the lever door and foam pad are intended to shield the operator from the UV activation and to apply a mild pressure on the slides, which facilitates the curing process.

A custom platform supports the glass slides in the cryostat and is used in the curing process. The slides are placed side-by-side, each into a slot in the top surface of the platform. Each slot is centered above an array of 65 UV-LEDs (Nichia Corp, NSPU510CS) soldered onto a four-layer printed-circuit board (PCB) (Sunstone Corporation). The platform contains a lever door, which is used to isolate the irradiated area and to protect the operator. An exploded view of the platform is shown in Fig 2. The Base Enclosure, the Lever Door and the Top-Cover were 3-D printed (PolyJet Technology) in VeroWhite, through Vistatek Corporation (Vadnais Heights, MN) and assembled with other off-the-shelf components. The model files and engineering drawings can be provided upon request.

#### 2.1.3 UV-LED array

The UV-LEDs are arranged in 5 rows of 13 LEDS, in a parallel resistor network, such that even UV intensity can be applied across the surface of the slides. The common terminals of the two arrays are connected to a single DC power source (Omron Corporation, S8VS-03005) and regulated by an on-off latching switch.

#### 2.1.4 Hand Rollers

The system requires the use of two different rollers. The hard-roller is composed of a hollow PVC pipe that is suspended on a plastic handle (Leica Corp, 39475218), and helps in applying adequate pressure onto the tape. This ensures a firm adhesion to the blockface of the sample. The soft-roller is made by threading a silicone tube over the PVC pipe of the hard-roller. This helps to remove air bubbles trapped between the glass slide and the tape-window, without damaging the tissue sections. The soft-roller helps to spread the applied pressure and prevent cracking of the sections.

#### 2.1.5 Tape-Windows and Slide Preparation

Standard adhesive tape-windows (Leica Corp, 39475214) were used for all samples. Either pre-coated slides (Leica Corp, 39475208) or those prepared in-house were used. The in-house slide coating procedure used Solution A (Leica Corp, 39475270) to clean the slides and Solution B (Leica Corp, 39475272) to coat the slides. Solution B is the UV-activated polymer that adheres the cut sections onto the glass slides. This was derived from the CryoJane operating protocol [1], with consultation from Instrumedics Corporation. In terms of section quality, we have not found a significant difference between the two slide types. We prefer to coat the slides in-house due to bulk cost savings.

#### 2.1.6 Cryostat and Environmental Setup

A standard Microm HM550, with a disposable knife carrier and MX-35 low-profile knife (Thermo Scientific, 3052835) was used to section all samples. The specimen temperature of the cryostat was set to -14±2°C; the temperature of the chamber was set to -16±2°C. The temperature was adjusted based on section quality (See Pitfalls Section 2.5). The environmental conditions surrounding the cryostat were actively regulated, such that the temperature was 17–19°C and the humidity was 45–60%. To ensure optimal circulation of cooled air in the chamber, the cryostat was defrosted weekly.

### 2.2 Brain Freezing and Tape-Transfer Sectioning Workflow

**Fig 3:**
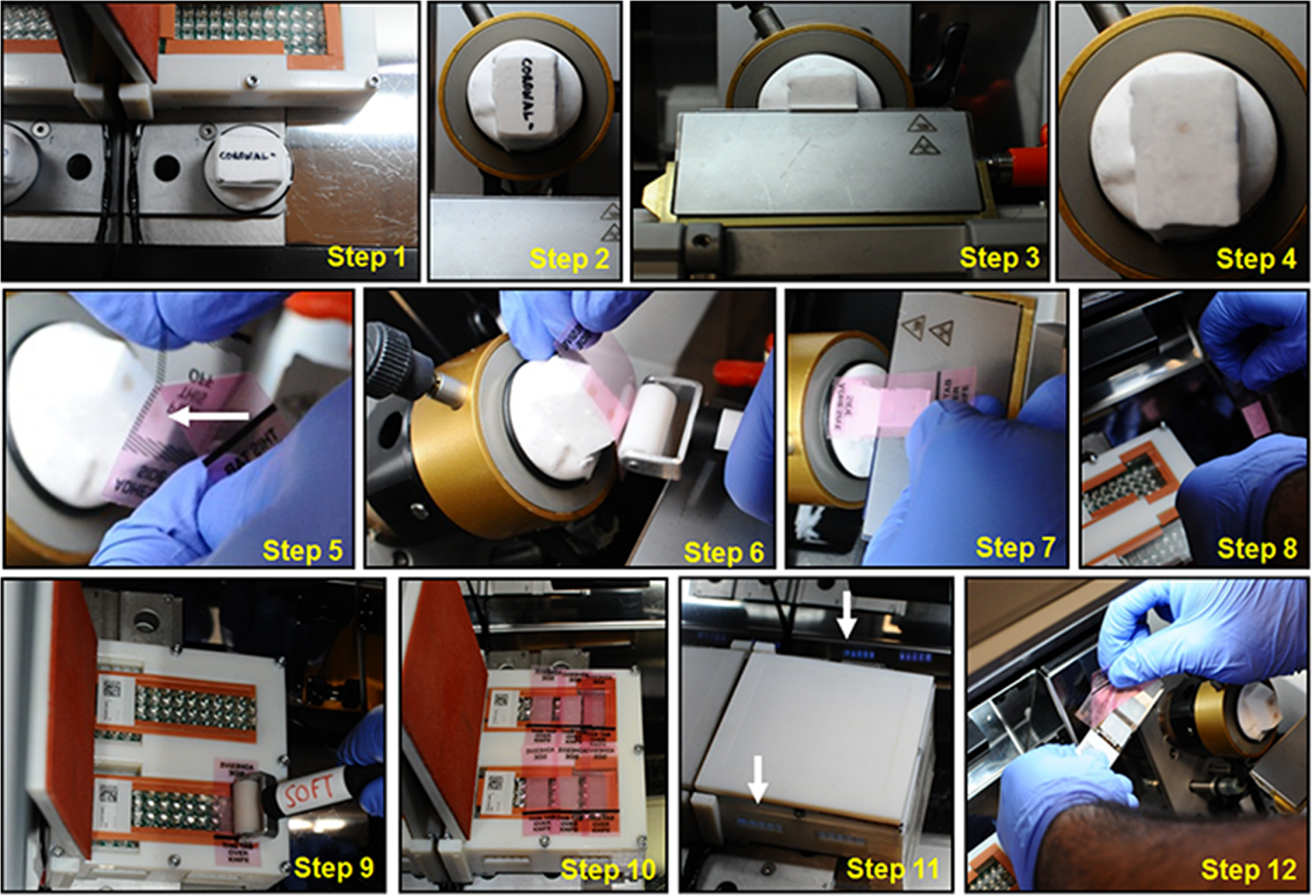
Production use of the tape-transfer system to section perfused mouse brains. This figure shows the use of this system to collect coronal sections of a perfused mouse brain. Step1, remove a frozen brain block from -80°C storage. Allow the block to acclimate in the cryostat for 30 minutes and then freeze the block onto the specimen chuck. The surface to be sectioned, should be facing up (towards the operator). Step2, attach the chuck and block to the specimen holder and orient relative to the knife. Step 3 and 4, trim the block at 50 or 100μm until the first indication of tissue. Step 5, peel the protective film (white arrow) from one chilled tape-window. Step 6, lightly attach the exposed adhesive surface of the tape-window to the blockface. The black line in the lower part of the tape-window should align with the bottom edge of the block. Use the hard-roller to further press and adhere the tape onto the block. Step 7, while supporting the bottom part of the tape-window, cut a 20μm section from the block. Step 8 and 9, move the tape-window and attached section, to the back of the cryostat and then onto a coated glass slide on the curing platform. Use the soft-roller to smooth the tape. Step 10, repeat Steps 5–9 until a total of 3 tape-windows are attached to each slide. Step 11, close the lever-door and apply the UV for 8–10 seconds. Reflections of the UV can be seen in the metal surface of the cryostat and through the open vents. Step 12, peel the tape-windows from each slide at a slight angle and remove the slides from the cryostat.

A 12-part illustration of the tape-transfer method is summarized in Fig 3. The method is described for the sectioning of a brain block of either one or two embedded mouse brains [2]. Dual-brain blocks were used for sectioning coronal samples; single-blocks were used for sagittal and horizontal sectioning. Single-brain blocks were also used for sectioning rat and macaque brains.

#### 2.2.1 Brain Freezing

Post cryo-protection, the brains were embedded in Neg-50 (Richard Allen Scientific, 6502) and frozen using dry-ice chilled isopentane at a temperature of approximately -60°C [2]. For single brain sectioning (1 brain per frozen block), standard cryostat embedding procedure was used [3]. To expedite the sectioning process, we also developed a dual-brain sectioning procedure (2 brains per frozen block), where custom brain-freezing molds were used [2]. Post-freezing, the brain block was trimmed using a razor to a width of ~ 20mm and a height of ~28mm.

#### 2.2.2 Brain block preparation (Fig 3, Steps 1–4)

In Step 1, a frozen tissue block is removed from -80°C storage, allowed to acclimate for 30 minutes in the cryostat, and then frozen onto the specimen chuck. The block is orientated, typically to allow for coronal sectioning of the embedded brains. The posterior part of the brain specimen is closest to the surface of the chuck. During this time, tape-windows and coated slides should be placed into the cryostat for cooling.

Next (Step 2), the chuck and block are attached to the specimen holder and oriented relative to the knife. The specimen holder is then advanced toward the knife and the block is trimmed at 50 or 100μm until the first indication of tissue is visible (Steps 3 and 4). The block is now ready for sample collection using the tape method.

#### 2.2.3 Section collection onto tape (Fig 3, Steps 5&6)

One chilled tape-window is peeled to remove the center protective film (Step 5) and the exposed adhesive surface is lightly attached by hand to the blockface. The black line in the lower part of the tape-window should align with the bottom edge of the block. The hard-roller is then used to further press and adhere the tape onto the block (Step 6).

#### 2.2.4 Section transfer to slide (Fig 3, Steps 7–10)

In Step 7, while the operator supports the bottom part of the tape (the portion marked “THIS TAB OVER KNIFE”), a 20μm section is cut from the block. The combination of tape + section is then quickly moved into the cold stream of the cryostat (back of the cryostat) and placed onto a coated glass slide on the curing platform (Steps 8 and 9). The soft roller is used to smooth the tape. This process is repeated for 3 tape-windows per slide. Alternating sections are commonly separated into two slide series for different histological procedures (Step 10).

#### 2.2.5 UV curing of the sections (Fig 3, Steps 11&12)

The lever-door of the curing platform is lowered and a pulse of UV is applied for 8–10 seconds (Step 11). Blue light is seen in the vents of the curing platform. The lever-door is opened and the tape-windows of each slide are peeled at a slight angle (Step 12). The slides are than removed from the cryostat and are ready for staining and coverslipping.

### 2.3 Histology and Imaging

The sectioned material was allowed to dry overnight at 4°C. Sections were stained for Nissl bodies, using a thionin based protocol, and coverslipped using DPX. Sections from AAV-injected and transgenic animals (see below) were briefly dehydrated in grades of ethanol (50, 75 and 90%) and coverslipped using DPX. To demonstrate compatibility with a range of common histochemical methods, sections were processed for BDA [4], Wisteria Floribunda Lectin (WFA) [5], Tyrosine Hydroxylase [6] and myelin [7]. In all cases, staining was performed on-the-slide, using either the LabVision 720 (Thermo Scientific) or sealed staining trays (StainTray, M920-2). Subsequent imaging and digitization of the sections were performed using a NanoZoomer HT system, at a resolution of 0.5μm/pixel. For fluorescent imaging, a tri-filter cube (DAPI-FITC-Texas Red filter) (Olympus, L10387) was used.

### 2.4 Stereotaxic Injections

To show that tape-transfer cut sections are compatible with tract tracing, a subset of animals was injected with anterograde or retrograde tracers. Survival surgery was carried out according to NIH guidelines and protocols approved by the CSHL IACUC. Vaporized isoflurane (1.5%) was used as the anesthetic. In some animals, two injections of adeno-associated virus (AAV) were performed per animal, the first with AAV-GFP (AAV2/1.CB7.CI.EGFP.WPRE.RBG) and the second with AAV-RPF (AAV2/1.CAG.tdTomato.WPRE.SV40). Both versions were obtained from the University of Pennsylvania vector core. Other animals were injected with 10% biotinylated dextran amine (BDA) (Invitrogen, D1956). In all cases, the Nanoject II (Drummond, 3-000-204) was used to inject a target volume of ~10nL. The AAV-injected animals survived four weeks post-injection, and the BDA-injected animals, for one week. They were then deeply anesthetized and perfused as above (2.1.1).

### 2.5 Pitfalls

The tape-transfer method requires more rigorous controls than conventional sectioning, but when standardized, is very reliable. The major sources of potential error are described below:

#### 2.5.1 Environmental Temperature and Humidity

More so than conventional cryostat sectioning, the tape-transfer method requires a stable chamber temperature. We have found that if the outside temperature is above 22°C then the cryostat has difficulty in regulating chamber temperature. For this reason, we maintained the room temperature to 17–19°C. The chamber temperature must remain 3–4°C colder than the specimen temperature. This will ensure that the tape-windows and the slides are also colder than the specimen. If a tape-window warms while adhered to the block or the cut section, the tissue will immediately warm. This warming will cause the section to compress. Since the section is attached to the tape, the adhesive will counteract this force, resulting in wide-spread tearing. Until the sections are UV cured onto the slide, the temperature of the section and any surface that it interacts with, must not rise above the specimen temperature.

We have also found that periods of high-humidity can cause excessive ice buildup in the cryostat, which can hamper air flow and cause the chamber to warm. This is most prevalent in the summer months, when we had to place a dedicated dehumidifier (Frigidaire, FAD504TDD) alongside the cryostat. Even at nominal external humidity, we have found that defrosting the cryostat weekly leads to more consistent section quality.

We have also found that if the humidity drops below 35%, tissue sections are prone to rapid dehydration (while attached to the tape) and can fracture. This is most prevalent in the winter months, when we placed a dedicated humidifier (Honeywell, HCM-6009) alongside the cryostat.

#### 2.5.2 Other Sources of Thermal Damage

When the section is adhered to tape, it is most sensitive to thermal damage. This damage is commonly caused by poor airflow (as discussed above), or interaction with the warm skin of the operator. It is important that the operator never touches the center surface of the tape window; the tape should only be handled by the unpeeled top and bottom tabs (marked: “THIS TAB OVER KNIFE”). It also is important to guard against warm air currents in the front of the chamber. We advise minimizing opening of the chamber lid and quickly moving the cut section, once cut, to the back to the cryostat. Finally, prior to section collection, it is important to chill both the tape-windows and the slides in the cryostat.

#### 2.5.3 Section Peeling

The UV activation of the polymer-coated slides is temperature sensitive. The slides should be chilled in the cryostat for no more than 30 minutes, otherwise the effectiveness of the applied UV activation will diminish and the sections will not adhere properly to the slide. It is also important to regularly clean the glass surface of the curing platform with absolute alcohol to remove contaminants such as frost and dust–this will ensure maximum passage of the applied UV light.

#### 2.5.4 Tissue Shear

We have found that unless the embedding medium freezes to the same hardness as the tissue, tissue sections are likely to have shear artifacts. These will appear as local or global tears in the tissue. We have empirically found that Neg-50 (Richard Allen Scientific, 6506) outperforms O.C.T (Tissue-Tek, 4583) or other embedding mediums. With the use of Neg-50, this artifact has been largely eliminated.

#### 2.5.5 Application of UV

This system allows the user to select the duration of UV exposure per slide. For most samples, an exposure of 5 seconds will be sufficient. We suggest an exposure of 8–10 seconds for maximum consistency. An exposure of longer than ~30 seconds will make it difficult to peel the tape-window, and should be avoided. If tissue remains on the tape-window after peeling, exposure should be increased by a few seconds. If excessive force is needed to peel the tape-window, then the exposure should be reduced.

## Results

**Fig 4:**
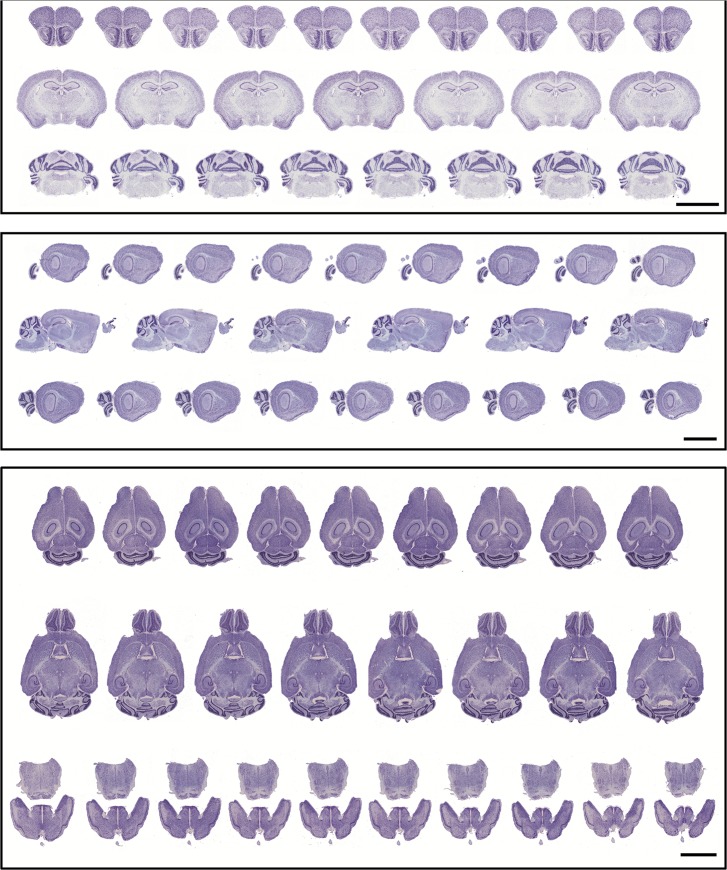
Thumbnail images of serial sections, cut from a perfused mouse brain in three standard sectioning planes. Selected rows of serial sections cut in the coronal (top), sagittal (middle), and horizontal (bottom) planes. Sections were collected using the tape-transfer method, processed for Nissl stain, coverslipped, and imaged. In all cases, the sections appear of consistently high-quality. A detailed examination of all sections from these three series is shown in Table 2.

**Fig 5:**
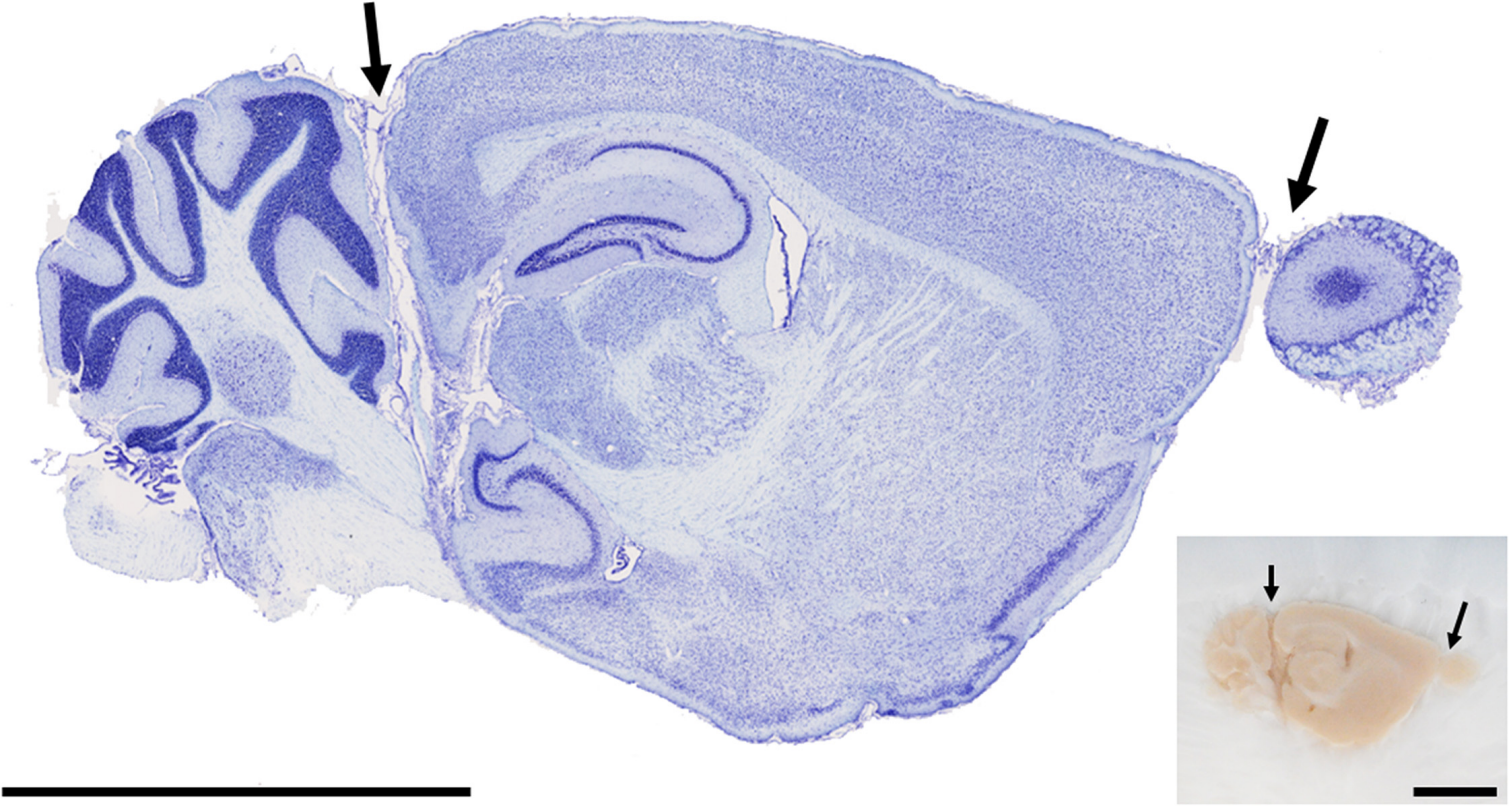
Tape-transfer cut sections are directly congruent to the blockface. A sagittal Nissl-stained section (approx. Lateral 1.725 mm) collected by the tape-transfer method. Arrows indicate separated pieces of brain (cerebellum and olfactory bulb) which nevertheless remain in an intact anatomical relationship, as in the original whole brain. Compare orientation with the inset of the frozen blockface at lower right (small arrows mirror large arrows in the histological section). Scale bars are 5mm.

The main point of the tape-transfer method is to assure excellent section quality, as judged by the final histological preparations. As shown in Fig 4, serial sections cut in the coronal, sagittal, and horizontal planes are of uniform and consistent high quality, such that only ~12–15% of the captured sections have blemishes or damage (Table 2). We consider that an average damage rate of 12–15% damage is very positive, especially since every 20μm section is collected and evaluated. Taking the Allen Reference Atlas as a comparison, our group routinely collects 4X more coronal and 20X more sagittal sections, but without an increase in the damage rate. Also to be noted, the tape-transfer method helps to preserve the section as it appears in the blockface (Fig 5). That is, disjointed pieces of tissue are transferred directly to the slide and preserved in their original spatial relationship.

**Table 2:** Rate of various tissue defects compared between Tape-Transfer sectioned material and published images from the Allen Reference Atlas.

**Brain Damage Type**	Coronal	Sagittal	Horizontal	Coronal ARA	Sagittal ARA
**Torn and Folded Section**	46	30	37	14	2
**Knife**	15	5	5	0	0
**Mechanical Cellular**	0	0	0	0	0
**Thermal (Cortical)**	4	10	2	0	0
**Thermal (Sub-Cortical)**	1	0	0	2	1
**Tissue Cracking**	0	0	0	0	0
**Entire Section Missing**	0	0	1	0	0
**Imaging Artifacts**	0	10	0	0	0
**Total Damaged Sections**	66	55	45	16	3
**Total Mounted Sections**	570	387	300	132	20
Percent Damaged	12%	14%	15%	12%	15%

Most of the categories are self-explanatory. Those that are not, we define as such: "Knife Damage" refers to torn sections that display a periodic "window blind" like pattern parallel to the knife. "Entire Section Missing" refers to instances where the section was intended to be collected, but did not adhere to the slide. “Thermal Damage” refers to non-anatomical holes that can appear in the tissue due to a rapid change in Temperature of the sections during cutting. Whole-brain thermal damage can also be caused due to improper cryo-protection of the sample during preparation. For the Allen Reference Atlas brains, only the sections shown in the atlas have been evaluated. For the coronal brain, these represent ¼ of the collected 25μm sections (~100μm spacing). Scale bars are 5mm.

**Fig 6:**
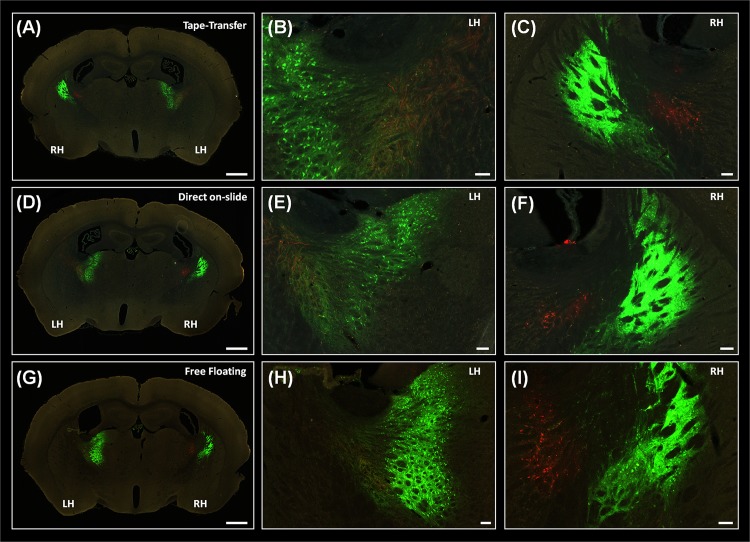
Compatibility of tape-transfer and the detection of injection GFP/RFP signal, compared to conventional sectioning methods. Coronal sections of a perfused mouse brain, that was injected with AAV-GFP and AAV-RFP and cut using tape-transfer (A-C); direct on-slide (D-F) and free-floating (G-I) technique. Low-magnification view of each section is shown in A, D and G. Sections correspond to ARA coronal levels 66 to 67.Higher-magnification view of the labeled cells and fibers in the left-hemisphere is shown in B, E, H and in the right-hemisphere in C, F, I. The sections are shown as they appear on the slide, without any adjustment. No major difference in fluorescence intensity is evident, for either the AAV-GFP or AAV-RFP. Note, the tape-transfer cut section (A-C) appears in a different left-right orientation than the other two sectioning modes. This is because the tape-transfer method preserves the orientation of the section as it appears on the blockface (Fig 5). The notation LH and RH is used to indicate the left or right hemisphere, as it relates to the brain. Scale bars are: 1mm (A, D, G) and 100μm (B, C, E, F, H, I).

To evaluate tape-transfer sectioned material relative to the conventional methods of collecting cryostat cut sections, we serially sectioned an AAV-injected brain and collected sections by (1) the tape-transfer method, (2) directly on the slide and (3) by brush for the free-floating method. Direct on-slide sections were cut with the anti-roll plate [8]. Free-floating sections were collected using a camel-hair brush and transferred into a 12-well plate, filled with PBS. The sections from both methods were mounted onto Superfrost (Fisher Scientific, 22-035-813) slides. The same staining and imaging conditions were used for all samples. The quality of signal was comparable in all three methods, but the consistency of histological sectioning was superior with the tape-transfer method (Fig 6). Note that the tape-transfer sections have a left-right orientation that directly corresponds to the brain. With free-floating sections, the consistency of left-right orientation on the slide is harder to maintain; and with direct on-slide sectioning, there is often a mirror flip for each section.

**Fig 7:**
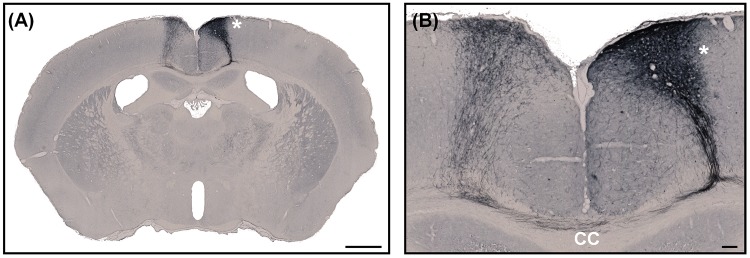
Histochemical detection of BDA sections, cut using the tape-transfer system. A coronal section (approx. Bregma -1.055 mm) of a perfused mouse brain, with a cortical injection of BDA (asterisk). The contralateral projections can be seen in clear detail at higher-magnification in B. The staining is of high-quality and labeled fibers are obvious in the corpus callosum (CC) and in the projection site. Scale bars are: 1mm (A) and 100μm (B).

**Fig 8:**
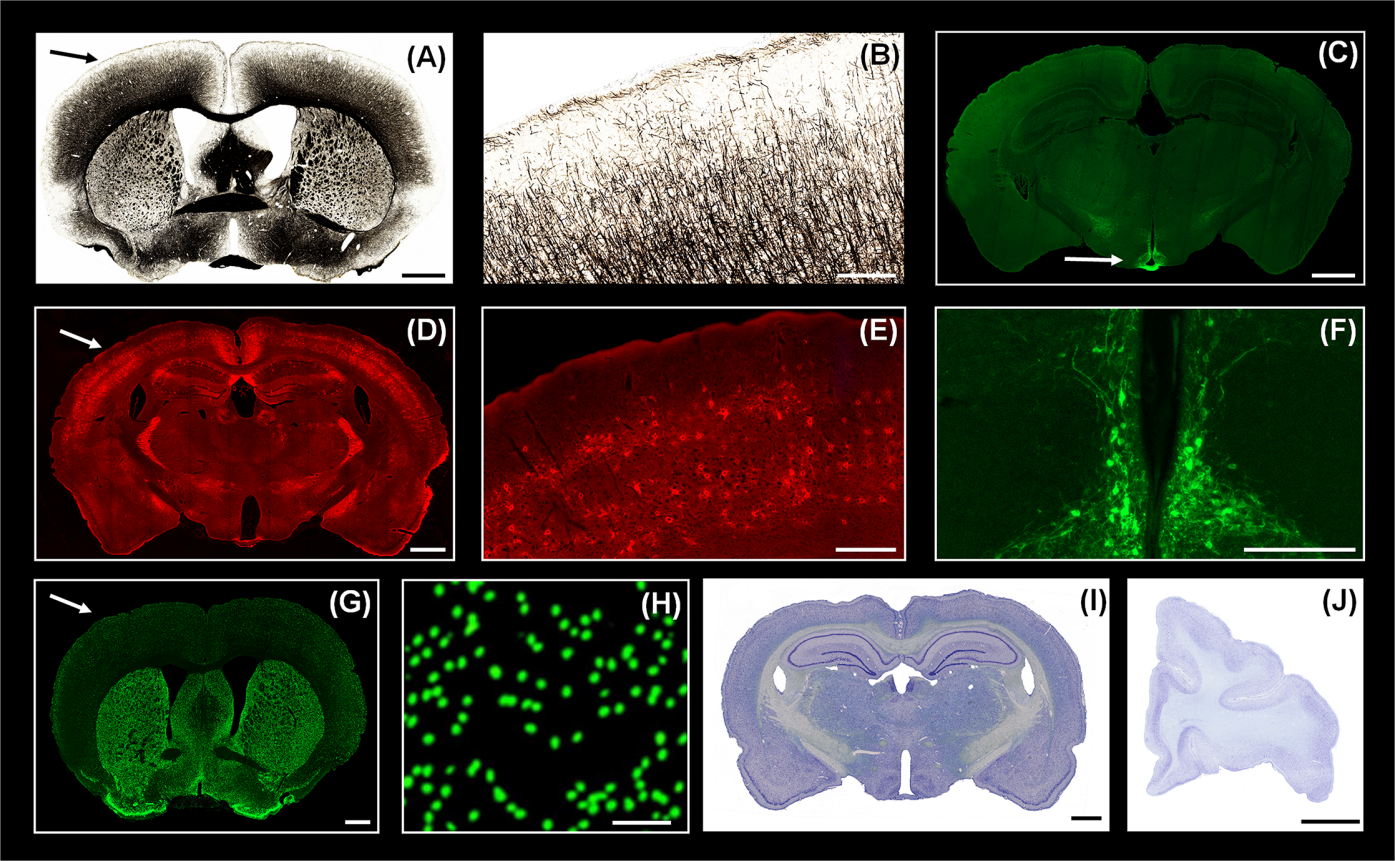
Histochemical, Immunohistochemical staining and the use of tape-transfer to section rat and macaque tissue. Low-magnification views of coronal sections, from mouse brains, reacted for (A) myelin Gallays silver stain technique), (C) tyrosine hydroxylase (TH), and (D) wisteria floribunda lectin (WFA). For each image, the arrow points to the region shown at higher magnification in (B), (E), and (F). Tissue with native fluorescent expression is shown in (G) (and, higher magnification, H) for a mouse brain expressing Cre-dependent GFP in GAD2 cells. Coronal sections of rat and monkey brain (frontal cortex of one hemisphere, medial surface at the left), collected by the tape-transfer method are shown in (I) and (J). All sections are 20μm thick. Scale bars are: 1mm (A, C, D, G, I); 200μm (B, E, F); 50μm (H); 6mm (J). The stereotaxic coordinates of the low-magnification sections are relative to Bregma (Allen Reference Atlas): +.145mm (A); -2.055mm (C); -1.555 (D); +.545mm (G); -2.85 ([12] (I)).

As shown in Fig 7 and Fig 8, tape-transfer sections can also be used for a range of histochemical processing: detection of injected BDA, myelin staining, immunohistochemistry for Wisteria Floribunda Lectin (WFA) or Tyrosine Hydroxylase (TH). We have also established that the method is suitable for sectioning rat (male Long–Evans rats) and rhesus macaque tissue, to the same level of quality as the smaller mouse brain. For the macaque, we blocked the brain to the approximate size of the mouse brain block (~20X28mm), so that the same tape-windows and 1X3 slides could be used.

## Discussion

We have presented a modification tape-transfer system, which allows for effective sectioning of perfusion fixed tissue, without a sacrifice in the sectioning rate or tissue quality. A frozen block can be cut and mounted on glass as serial 20μm sections in about 4 hours (~600 sections). For coronal sectioning, where two brains can be frozen in one block [2], the sectioning rate per brain is effectively halved to 2 hours. One distinct advantage is the time saved from brush-mounting of free-floating sections. Another advantage is the quality of the histological sections, in terms of freedom from folding or tears, and the faithful preservation of the inherent gross anatomy (Fig 4). Left-right orientation of the brain is automatically achieved, without the need of a pinhole or other fiducial mark, and the need to “re-position” dissociated brain segments is eliminated (Fig 5). This feature becomes important in applications requiring exact alignment of consecutive sections. For on-the-slide histochemistry, the tape-transfer system has the additional advantage of a superior adhesion between the section and slide. This greatly reduces the number of sections lost during agitation or solution changes.

The redesigned system is an improvement on the commercial CryoJane tape-transfer system. The redesign is centered on the use of an array of UV-LEDs to cure the section on the slide. To compensate for the low nominal irradiance of each LED, we increase the application time from 8 milliseconds for CryoJane to around 5–6 seconds. The overall UV irradiance of the slide is around 50X higher than in a single pulse of the CryoJane system. This increase in irradiance ensures that the sections are firmly adhered to the slide and reduces the risk that a piece of the section is peeled away. We also designed the UV-array such that it evenly applies the UV energy across the entire slide–this allows us to maximize the amount of tissue on the slide. The redesign features two curing stations, instead of one, and this allows for the convenient collection of alternating sections in two slide series. In addition to the hardware improvements, we have optimized: (1) the conditions for cryo-protection, (2) the choice of embedding medium (3) the environmental conditions inside of the cryostat (4) the use of the soft-roller to smooth the tape and sections on the coated glass slide.

The tape-transfer method is most applicable to three user cases: (1) non-expert cryostat users, such as students; (2) low-volume production of high-quality sections, (3) high-volume production of thin (<50μm) serial sections, such as for 3-D digital reconstruction. The tape-transfer method eliminates the need for the operator to directly handle and mount tissue sections on the slide. Instead, the operator uses the adhesive tape to ferry the sections onto the slide. This allows even novice users to quickly become proficient in collecting high-quality sections and helps to reduce variations in tissue quality across operators.

Our primary use of the tape-transfer system has been for the collection of 20μm sections, but sections of other thicknesses can also be collected. In our experience, the system is most appropriate for 10–50μm thick sections; 50–100μm thick sections can be collected, but these are difficult to stain on-the-slide. We emphasize the processing of perfused tissue, because to our knowledge, tape-transfer method has previously been used primarily for the sectioning of fresh-frozen tissue or bone [9–11]. Perfused tissue is more challenging to section using the tape-transfer system, because of the need to consider the interaction between the cryoprotectant (sucrose) and the adhesive tape.
